# Identification of the *Streptococcus mutans* LytST two-component regulon reveals its contribution to oxidative stress tolerance

**DOI:** 10.1186/1471-2180-12-187

**Published:** 2012-09-01

**Authors:** Sang-Joon Ahn, Ming-Da Qu, Elisha Roberts, Robert A Burne, Kelly C Rice

**Affiliations:** 1 Department of Oral Biology, College of Dentistry, University of Florida, Gainesville, FL 32611, USA; 2 Department of Microbiology and Cell Science, College of Agricultural and Life Sciences, University of Florida, Gainesville, FL 32611, USA

**Keywords:** Stress, Oxygen, Competence, Cid/Lrg system, *Streptococcus mutans*

## Abstract

**Background:**

The *S. mutans* LrgA/B holin-like proteins have been shown to affect biofilm formation and oxidative stress tolerance, and are regulated by oxygenation, glucose levels, and by the LytST two-component system. In this study, we sought to determine if LytST was involved in regulating *lrgAB* expression in response to glucose and oxygenation in *S. mutans*.

**Results:**

Real-time PCR revealed that growth phase-dependent regulation of *lrgAB* expression in response to glucose metabolism is mediated by LytST under low-oxygen conditions. However, the effect of LytST on *lrgAB* expression was less pronounced when cells were grown with aeration. RNA expression profiles in the wild-type and *lytS* mutant strains were compared using microarrays in early exponential and late exponential phase cells. The expression of 40 and 136 genes in early-exponential and late exponential phase, respectively, was altered in the *lytS* mutant. Although expression of *comYB*, encoding a DNA binding-uptake protein, was substantially increased in the *lytS* mutant, this did not translate to an effect on competence. However, a *lrgA* mutant displayed a substantial decrease in transformation efficiency, suggestive of a previously-unknown link between LrgA and *S. mutans* competence development. Finally, increased expression of genes encoding antioxidant and DNA recombination/repair enzymes was observed in the *lytS* mutant, suggesting that the mutant may be subjected to increased oxidative stress during normal growth. Although the intracellular levels of reaction oxygen species (ROS) appeared similar between wild-type and *lytS* mutant strains after overnight growth, challenge of these strains with hydrogen peroxide (H_2_O_2_) resulted in increased intracellular ROS in the *lytS* mutant.

**Conclusions:**

Overall, these results: (1) Reinforce the importance of LytST in governing *lrgAB* expression in response to glucose and oxygen, (2) Define a new role for LytST in global gene regulation and resistance to H_2_O_2_, and (3) Uncover a potential link between LrgAB and competence development in *S. mutans*.

## Background

*Streptococcus mutans* is considered the primary causative agent of dental caries, and when transiently introduced into the bloodstream following daily dental hygienic practices such as toothbrushing and flossing, this bacterium can also cause potentially lethal infective endocarditis (IE) [1-4]. In both infectious scenarios, the virulence of *S. mutans* depends upon its ability to form biofilms and to withstand extreme changes in environmental conditions, including fluctuations in oxygenation, shear stress, as well as nutrient source and availability. For example, in the oral cavity, *S. mutans* must be able to rapidly alter its expression of transporters and metabolic enzymes to catabolize a variety of host-derived dietary carbohydrates. Internalized carbohydrates are metabolized through the glycolytic pathway, resulting in the accumulation of acidic end-products in the environment, which favors the growth of *S. mutans* and other acid-tolerant cariogenic species. Repeated cycles of acidification can lead to a net demineralization of tooth enamel and the development of caries. Sucrose, a common dietary sweetener, can also be utilized by *S. mutans* for the production of extracellular polysaccharides [5-8] that facilitate bacterial adhesion and biofilm formation. Aeration has also been found to have a profound effect on carbohydrate metabolism and biofilm formation by *S. mutans*[9-11]. It is therefore not surprising that there is overlap in the genetic regulatory circuits responsive to carbohydrate metabolism, aeration/oxidative stress resistance and control of biofilm formation in *S. mutans*, which include CcpA [12-14], Rex [15], and Frp [16].

More recently, an emerging trend in the study of bacterial biofilms has been a focus on the contribution of bacterial cell death and autolysis to biofilm adherence, maturation, and dispersal. It has been demonstrated in a wide variety of bacteria that death and lysis of a subpopulation of cells can facilitate biofilm formation due to the release of DNA into the extracellular environment (eDNA) [17-22]. Likewise, cell death and lysis have been implicated in dispersal of cells from a mature biofilm [23-25]. In *Staphylococcus aureus*, the Cid/Lrg system has been shown to be involved in the regulation of cell death, autolysis, and biofilm formation [17,21,26-28]. Characterization of *S. aureus cid* and *lrg* mutants has revealed that these operons have opposing effects on cell death and murein hydrolase activity [27,29]. These observations, combined with the fact that LrgA and CidA share structural features with the bacteriophage lambda family of holin proteins [29], have led to the hypothesis that CidA and LrgA control cell death and lysis in a manner analogous to effector and inhibitor holins, respectively [26,30]. Bacteriophage holins are small membrane proteins that oligomerize in the cell membrane, acting as “molecular clocks” that regulate the timing and lysis of the host cell during lytic infection [31]. For example, the lambda S holin regulates cell death and lysis by the formation of large lipid-excluding “rafts” that promote cytosolic leakage as well as access of the phage-encoded endolysin (murein hydrolase) to the cell wall [32-34]. *S. aureus* CidA and LrgA have recently been shown to oligomerize into high-molecular-mass complexes in a cysteine disulfide bond-dependent manner, a biochemical feature also shared with holin proteins [35]. Although the molecular details of how Cid and Lrg function to control cell death and lysis have not yet been completely elucidated, the fact that *cid* and *lrg* homologues have been identified in a wide variety of bacterial and archeal genomes supports a fundamental and conserved role for this system in cell physiology [30,36].

In previous work it was determined that expression of potential *cidAB* and *lrgAB* homologues in *S. mutans* is highly responsive to carbohydrate availability [12,37] and oxygenation [11]. Given the potential importance of these genes to biofilm development in *S. mutans*, we previously characterized a panel of *S. mutans cid* and *lrg* isogenic mutants and found that a subset of these genes did indeed influence biofilm formation, production of glucosyltransferases (enzymes that synthesize extracellular glucan polymers that contribute to biofilm adhesion), and oxidative stress tolerance [37]. In this study it was also found that, as demonstrated previously in *S. aureus*[38,39], the *S. mutans* LytST two-component system was required for activation of *lrgAB* expression, but not *cidAB* expression [37]. Genes homologous to *lytST* appear to be present in most Gram-positive organisms that contain *lrgAB*[30] and these genes are often linked to one another, inferring an important role for this two-component system in fine-tuning *lrgAB* expression in response to external environmental signals. Therefore in this study, we sought to determine if LytST is involved in regulation of *lrgAB* expression in response to glucose and oxygenation in *S. mutans*, and to elaborate on the contribution of LytST to cellular homeostasis and global control of gene expression.

## Results

### Effects of oxygenation and glucose metabolism on *S. mutans lrg* and *cid* expression

The LytST two-component regulatory system has been shown to positively regulate *lrgAB* expression in a wide variety of bacteria, including various staphylococcal [38-40] and *Bacillus* species [41,42], as well as in *S. mutans*[37]. The conserved nature of this regulation in Gram-positive bacteria, combined with the known effects of LytST and LrgAB on cell death/lysis [29,38,39,43], biofilm development [21,37,38], and oxidative stress resistance [37], suggests that LytST and LrgAB are central regulators of physiologic homeostasis. However, little is known about the environmental and/or cellular cues to which LytS responds. In *S. aureus* and *B. anthracis*, it has been shown that *lrgAB* expression is responsive to disruption of cell membrane potential in a LytST-dependent manner [41,44]. However, we were unable to determine whether this regulation also occurs in *S. mutans*, as treatment with membrane-potential disrupting agents (gramicidin, carbonyl cyanide m-chlorophenylhydrazone) did not have a measurable effect on membrane potential, as assessed by staining with DIOC_2_ (3) (data not shown).

**Figure 1:**
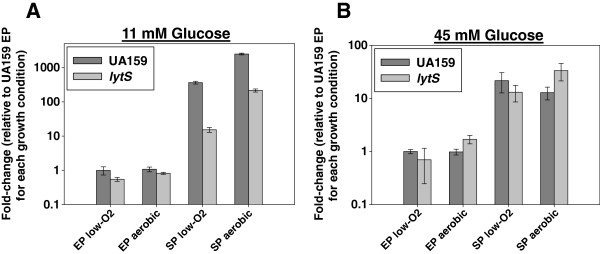
**LytS-dependent expression of *lrgAB *in *S****.****mutans*. **Overnight cultures were diluted in THYE, containing either 11 mM (**A**) or 45 mM glucose (**B**) to an OD_600_ = 0.02 and grown at 37°C as static cultures at 5% CO_2_ (“low-O_2_”) or as aerobic shaking cultures at 250 RPM (“aerobic”). RNA was harvested at exponential (EP) and stationary phase (SP). Reverse-transcription, real-time PCR reactions, and determination of copy number were performed as described previously using *lrgA* and 16S-specific primers [37,77]. Fold-change expression of *lrgAB* and 16S under each growth condition was calculated by dividing the gene copy number of each test sample by the average gene copy number of UA159 EP. Data was then normalized by dividing each *lrgAB* fold-change value by its corresponding 16S fold-change expression value. Data represent the average of 3 biological replicates. Dark grey bars represent UA159 and light grey bars represent *lytS* mutant. Error Bars represent the standard error (SEM).

In previous studies, it was shown that oxygen and glucose metabolism have a pronounced effect on *lrg* and *cid* expression in *S. mutans*, but the specific role of LytS, if any, in this regulation was not addressed [11,37]. Therefore, *S. mutans* UA159 and its isogenic *lytS* mutant were grown under aerobic and low-oxygen conditions to exponential (EP) and stationary (SP) growth phases in media containing 11 mM or 45 mM glucose. Quantitative real-time reverse transcriptase PCR (qRT-PCR) was performed on RNA isolated from cultures at each time point to assess changes in *lrg* expression (Figure 1). In UA159, stationary phase *lrgAB* expression was upregulated 365-fold relative to exponential phase when grown under 11 mM glucose and low-oxygen conditions (Figure 1A)*.* Although mutation of *lytS* resulted in a severe loss of stationary phase *lrgAB* induction in cells grown in 11 mM glucose, *lrgAB* expression was not completely abolished. When grown under aerobic conditions and 11 mM glucose, stationary phase *lrgAB* expression was upregulated 2500-fold relative to exponential phase in the wild-type strain (Figure 1A), confirming previously-published observations that aerobic growth promotes *lrgAB* expression [11]. However, stationary-phase *lrgAB* expression was still induced 216-fold in the *lytS* mutant during aerobic growth, suggesting that (1) other as-yet-unknown regulators also contribute to the positive control of *lrgAB* expression during aerated growth, and (2) LytST is a predominant regulator of *lrgAB* expression during low oxygen growth, compared to aerobic growth. Under low-oxygen and aerated cultures, stationary phase induction of *lrgAB* expression was dramatically reduced when grown in 45 mM glucose, and similar levels of expression were observed in the wild-type and *lytS* mutant (Figure 1B), suggesting that growth in high levels of glucose abrogates oxygen-dependent regulation of *lrgAB* by LytST. Consistent with previously-published data [37], LytS did not appear to have a measurable effect on *cidAB* expression under any of the growth conditions tested here (data not shown). In summary, LytST-dependent regulation of *lrgAB* expression is much more pronounced during low-oxygen growth and at low glucose levels.

### Microarray analysis of the LytS regulon

**Figure 2:**
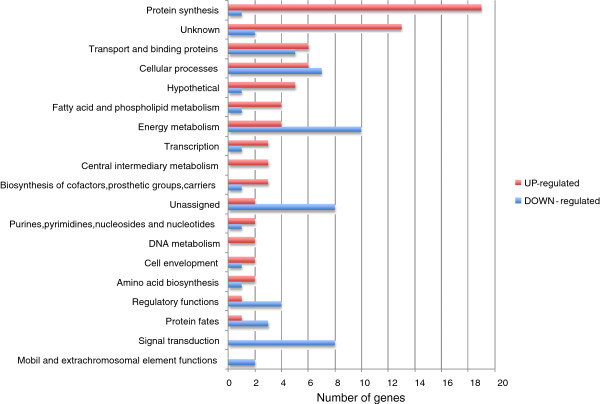
**Distribution of functions of genes affected by loss of LytS at late exponential phase.** Statistical analysis was carried out with BRB array tools (http://linus.nci.nih.gov/BRB-ArrayTools.html/) with a cutoff *P* value of 0.001. The 136 genes differentially expressed at *P* ≤0.001 are grouped by functional classification according to the Los Alamos *S. mutans* genome database (http://www.oralgen.lanl.gov/).

Based on the transcriptional data presented above, the effects of LytST regulation on *lrgAB* expression are most evident while *S. mutans* is growing under conditions of low-oxygen (5% CO_2_) with a lower concentration of glucose. To begin to explore how LytST impacts critical phenotypes of *S. mutans*, RNA expression profiles in UA159 and the *lytS* mutant were compared using an RNA microarray approach. RNA was isolated from early exponential and late exponential growth phases from static planktonic cultures grown in BHI (containing 11 mM total glucose) at 37°C in a 5% CO_2_ atmosphere (Additional file 1: Table S1 and Additional file 2: Table S2). At early exponential growth phase, loss of LytS affected the expression of 40 genes (12 upregulated and 28 downregulated; *P <* 0.005; Additional file 1: Table S1). Most of the upregulated genes in early exponential phase displayed only a modest increase in expression and included genes involved in DNA repair, purine/pyrimidine metabolism, competence, and a number of unassigned and hypothetical ORFs. RNA transcripts that were strongly down-regulated greater than 10-fold in cells lacking LytS during early exponential growth included those annotated as bacitracin/surfactin/gramicidin synthesis proteins, transport and binding proteins, and LrgAB. In contrast, loss of LytS affected the expression of a much larger number of genes in late exponential phase (136 genes total), with 79 upregulated transcripts and 57 downregulated transcripts (*P* < 0.001; Additional file 2: Table S2). Aside from dramatically decreased *lrgAB* expression, affected genes included those involved in amino acid and co-factor biosynthesis, carbohydrate and fatty acid metabolism, stress adaptation, toxin production, DNA repair/recombination, protein synthesis, transcriptional regulation, and competence, as well as multiple hypothetical and/or unassigned ORFs (Additional file 2: Table S2 and Figure 2). A subset of genes was differentially expressed as a function of the loss of LytS in both early exponential and late exponential growth phases (Additional file 1: Table S1 and Additional file 2: Table S2). These included many genes encoded by the *S. mutans* genomic island TnSMu2 [45] (SMU.1335c, 1339-1342, 1344c-1346, 1354c, 1360c, 1363c, 1366c), *ssbA*, *comYB*, and *lrgAB*. Given that these genes were regulated by LytS in both growth phases examined, it is possible that they are under the direct control of LytST. To validate the microarray data, qRT-PCR was performed on late exponential phase wild-type and *lytS* mutant RNA to assess expression of 14 of the affected genes. As shown in Table 1, the expression ratios (*lytS* mutant/wild-type) for each gene obtained by real-time PCR were similar to the microarray results. Interestingly, expression ratios of these genes were all close to 1.0 when comparing expression between the wild-type strain and a *lrgAB* mutant (Table 1), indicating that the differential expression patterns observed in the *lytS* mutant were not a consequence of down-regulated *lrgAB* expression.

**Table 1:** Real-time PCR validation of RNA microarray results

		**Microarray**	**Real-time pcr**		
		***lytS *mutant**	***lytS *mutant**	***lrgAB *mutant**	
(SMU.1985)	*comYA (comYB)*	22.9927	6.8449	0.8163	
SMU.1967	*ssbA*	5.5803	4.1076	0.8791	
(SMU.1515)	*vicR (vicX)*	2.6764	1.7647	1.0267	
SMU.924	*tpx*	2.4148	3.6168	1.058	
SMU.1739	*fabF*	2.2443	2.0333	1.084	
SMU.1666	*livG*	2.1183	3.4331	1.009	
SMU.80	*hrcA*	0.4953	0.6107	1.0204	
SMU.1424	*pdhD*	0.4769	0.4031	1.2004	
SMU.580	*xseA*	0.29849	0.5409	1.1398	
SMU.1600	*celB*	0.2186	0.2825	1.2979	
SMU.113	*pfk*	0.1597	0.176	1.3578	
SMU.82	*dnaK*	0.1523	0.2652	0.9907	
SMU.1344	*fabD*	0.0223	0.012	1.0637	
SMU.1341	*grs*	0.0008	0.0121	1.1027	

Results are expressed in fold-change (mutant/wild-type).

### Investigation of the effect of LytST and LrgAB on competence

**Figure 3:**
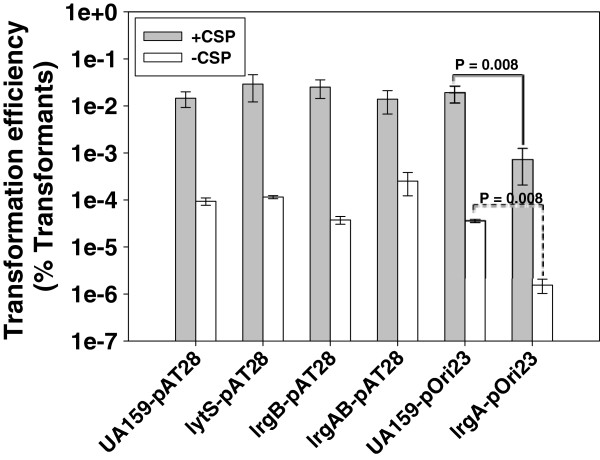
**Transformation efficiencies of UA159 and isogenic *lytS *and *lrg *mutants.** To compare the ability of UA159 and its isogenic *lytS*, *lrgA*, *lrgB*, and *lrgAB* mutants to take up exogenously-added plasmid DNA, a quantitative competence assay was performed on n = 4-6 biological replicates of each strain as described in Methods [83]. Plasmid pAT28 [encoding spectinomycin resistance; [84] was used to assess transformation efficiency in UA159, *lytS*, *lrgB*, and *lrgAB* mutants. Because the *lrgA* mutant was generated with a spectinomycin-resistance cassette [37], plasmid pORi23 [encoding erythromycin resistance; [85]] was used to assess transformation efficiency in UA159 and *lrgA* mutant. Transformation efficiencies (Y axis) in the presence (grey bars) and absence (white bars) of CSP are expressed as the percentage of transformants (CFU/ml on BHI + selective antibiotic) among total viable cells (CFU/ml on BHI). Error bars represent SEM. Brackets with *P* values denote statistically-significant differences between two samples (Mann–Whitney Rank Sum Test).

In analyzing the microarray data in Additional file 1: Table S1 and Additional file 2: Table S2, it appeared that the gene most highly upregulated in response to loss of LytS in both phases of growth was *comYB* (SMU.1985), a homologue of the *B. subtilis comGB* gene that encodes part of an ABC transporter essential for DNA binding-uptake during competence in *S. mutans*[46]. Interestingly, a *comYB* mutant of *S. mutans* was shown to be unaffected in competence signaling, but showed reduced biofilm formation, which was thought to be a function of its inability to bind biofilm matrix eDNA [47]. Since the *lytS* mutant displayed an increase in *comYB* expression (Additional file 1: Table S1 and Additional file 2: Table S2), we hypothesized that this strain may display alterations in its ability to form biofilm and/or its transformability during genetic competence. However, the *lytS* mutant did not display any appreciable difference in its ability to form static biofilm in the presence of glucose or sucrose (data not shown), and likewise, did not display a difference in its ability to uptake plasmid DNA in a quantitative competence assay, relative to the wild-type strain (Figure 3). Since *lrgAB* expression is so strongly regulated by LytST, the ability of isogenic *lrgA*, *lrgB*, and *lrgAB* mutants to uptake plasmid DNA via competence was also assessed (Figure 3). Of all the mutants tested, the *lrgA* mutant was the most severely impaired in its ability to uptake plasmid DNA relative to the parental strain, displaying a 26- and 24-fold decrease in transformation efficiency in the presence and absence of competence-stimulating peptide (CSP), respectively (Figure 3), suggesting that LrgA is somehow involved in genetic transformation in a CSP-independent manner. This finding has particular significance considering that LrgAB has been linked to regulation of cell death and lysis in *S. aureus*[21,29] and *S. mutans*[37], and these physiological processes are also extremely important during natural competence. It is interesting to note that, similar to the competence results described here, the *lrgA* mutant was previously shown to display decreased glucose-dependent biofilm formation and decreased glucosyltransferase production, whereas the *lrgB* and *lrgAB* mutants behaved in a manner similar to the parental strain [37]. These phenotypic patterns suggest that the presence of LrgB alone, rather than the lack of LrgA, may be responsible for the biofilm and competence phenotypes observed in the *lrgA* mutant.

### Effect of LytST on oxidative stress tolerance

**Figure 4:**
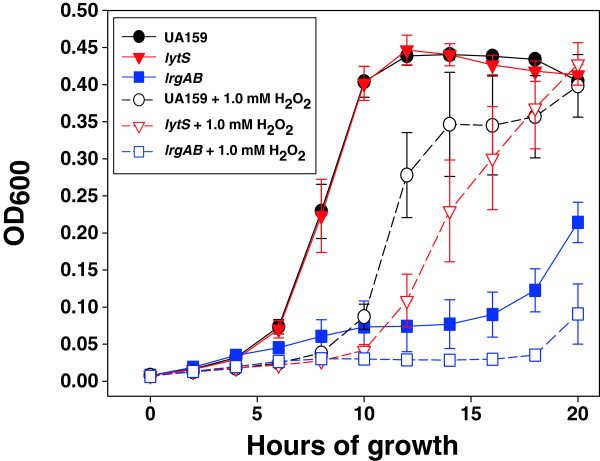
**H**_**2**_**O**_**2**_**challenge assay of UA159, *lytS *and *lrgAB *mutants.** Cultures of UA159, *lytS*, and *lrgAB* mutants (n = 6 biological replicates per strain) were grown in the presence (open symbols) and absence (filled symbols) of 1.0 mM H_2_O_2_ for 20 h at 37°C (aerobic atmosphere) in a Biotek microplate reader. OD_600_ measurements of each well were recorded at 2 h intervals. Black circles represent UA159, red triangles represent *lytS* mutant, blue squares represent *lrgAB* mutant. Error bars represent SEM.

Previously, our investigations disclosed a strong link between oxidative stress tolerance and the Cid/Lrg system [37], a role for these genes that had not been described in other organisms. Specifically, we found that *lrgAB*, *lrgB*, *cidAB*, and *cidB* mutants exhibited reduced growth in the presence of paraquat, and growth of *lrgAB*, *cidAB*, and *cidB* mutants on BHI agar plates in aerobic conditions was almost completely inhibited [37]. It is therefore interesting to note that in the *lytS* microarray results (Additional file 2: Table S2), genes encoding antioxidant and DNA repair/recombination enzymes were significantly upregulated in the *lytS* mutant in late exponential phase. These included *yghU* and *tpx*, encoding the putative anti-oxidant enzymes glutathione S-transferase and thiol peroxidase, respectively, as well as *recJ*, which encodes a single-stranded DNA exonuclease protein that facilitates DNA repair in response to oxidative stress [48-51]. To further investigate the effect of *lytS* and *lrgAB* on oxidative stress tolerance, wild-type, *lytS*, and *lrgAB* mutants were grown as planktonic static BHI cultures in aerobic atmosphere and in the presence and absence of H_2_O_2_ (Figure 4). When challenged with H_2_O_2_, UA159 experienced an increased lag phase of growth, and the overall OD of the culture was 10-25% less than the untreated culture until 20 h growth. Under these assay conditions, the *lrgAB* mutant displayed a dramatic growth defect in both the presence and absence of H_2_O_2_. It is interesting to note that this aerobic growth defect was also previously observed when the *lrgAB* mutant was grown in aerobic atmosphere on BHI agar plates [37]. The *lytS* mutant displayed an increased lag in growth relative to UA159 when cultured in the presence of H_2_O_2_, but OD values were comparable to the wild-type strain by 16 h growth. These results suggest that the LytST regulon impacts the ability of cells to grow under conditions of oxidative stress.

**Figure 5:**
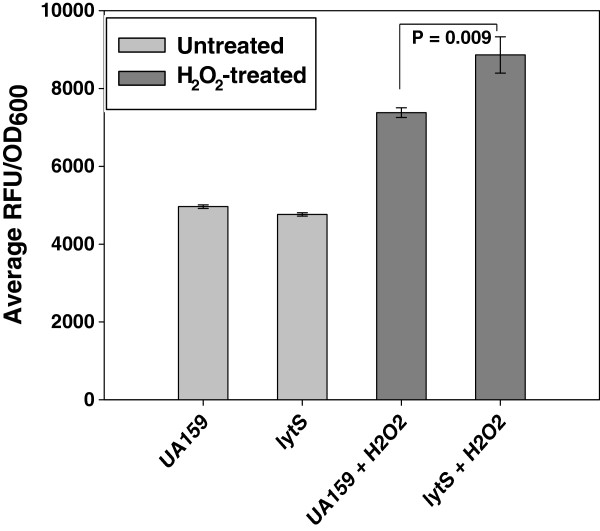
**Measurement of intracellular ROS in UA159 and *lytS *mutant by CM-H**_**2**_**DCFDA staining.** Cells were harvested from 20 h BHI cultures of UA159 and isogenic *lytS* mutant grown at 37°C 5% CO_2_ (n = 3-6 biological replicates each), resuspended in HBSS containing 5 μM CM-H_2_DCFDA, and incubated at 37°C to load the cells with stain. After 60 min incubation, cell suspensions were centrifuged, washed once in HBSS buffer, and then resuspended in HBSS buffer alone (light grey bars) or in HBSS containing 5 mM H_2_O_2_ (dark grey bars). Each suspension was transferred to wells of an optically-clear 96 well plate, and incubated at 37°C in a microplate reader. Cell fluorescence (as measured by relative fluorescence units; RFU) and the OD_600_ of each well was recorded after 30 min incubation. RFU measurements are expressed per OD_600_ of each well to account for any subtle variations in cell density. Error bars represent SEM. Brackets with *P* values denote statistically-significant differences between two samples (Mann–Whitney Rank Sum Test).

The cell-permeable fluorescent dye CM-H_2_DCFDA (Invitrogen Molecular Probes) was also used to assess intracellular ROS in UA159 and the *lytS* mutant (Figure 5). This fluorescent compound is oxidized in the presence of H_2_O_2_ and other reactive oxygen species (ROS) and is considered a general indicator of intracellular oxidative stress [52,53]. This analysis revealed that stationary-phase cultures of the wild-type and *lytS* mutant strains had similar “endogenous” intracellular levels of ROS (Figure 5, light grey bars). When stationary-phase cells from each strain were loaded with CM-H_2_DCFDA and then challenged with 5 mM H_2_O_2_ (Figure 5, dark grey bars), a greater increase in fluorescence was observed in the *lytS* mutant relative to UA159 (*P* = 0.009, Mann–Whitney Rank Sum Test), suggesting that loss of LytS has an impact on the ability of the cells to detoxify H_2_O_2_ and/or other intracellular ROS.

## Discussion

The transcriptome analyses presented in this study have revealed that the LytST two-component system has a widespread effect on gene expression in *S. mutans*. A much higher number of transcripts were affected by the *lytS* mutation in late exponential phase and the magnitude of changes in expression was greater (n = 136 genes, Additional file 2: Table S2) relative to early-exponential phase (n = 40 genes, Additional file 1: Table S1), where most genes exhibited only a modest (1-2 fold) change in expression. These differences in gene expression patterns are unlikely to be an indirect function of altered *lrgAB* expression in the *lytS* mutant, as expression of *lytS*-regulated genes was unaltered in an *lrgAB* mutant relative to the wild-type strain (Table 1). Taken together, these observations suggest that LytST exerts control over its transcriptome in a growth-phase dependent manner, and to our knowledge, this is the first study that has compared the scope of LytST regulation at different phases of growth. Interestingly, RNA microarray studies of *lyt* mutants have also been performed in *S. aureus*[38], *S. epidermidis*[40], and *B. subtilis*[42]. As we have observed here in *S. mutans*, a global effect of LytST on gene expression was also noted in *S. aureus* and *S. epidermidis*[38,40]. In *S. aureus*, LytST appeared to exert primarily positive effects on gene expression in exponential phase when aerobic cultures were grown in media containing excess (35 mM) glucose, as only 7 genes were found to be upregulated in the *lytS* mutant [38]. In *S. epidermidis*, a large number of genes were up- or down-regulated as a function of the presence of LytST during exponential phase during aerobic growth in medium containing 12 mM glucose [40]. In contrast, mutation of *lytS* only appeared to affect the expression of *lytST* itself and genes encoding *lrgAB* and *cidAB* homologues in *B. subtilis*[42]. However, due to the differences in growth conditions used (glucose levels and/or culture aeration) and the differing metabolic pathways present in these organisms, it is difficult to establish direct correlations between these studies and the *S. mutans* microarray results presented here.

As demonstrated previously [37], expression of *lrgAB* was also shown to be tightly controlled by the LytST two-component system in *S. mutans* in this study. Specifically, we have found that LytST-dependent expression of *lrgAB* is regulated in part by glucose metabolism and oxygen in *S. mutans* (Figure 1). Furthermore, control of *lrgAB* expression by LytST appears to be highly growth-phase dependent: *lrgAB* expression in the *lytS* mutant exhibited only a modest decrease in expression in early exponential phase (0.49 relative to UA159, Additional file 1: Table S1), whereas *lrgAB* expression was down-regulated some 200-fold in the *lytS* mutant at late exponential phase (Additional file 2: Table S2). Alternatively, it is possible that control of *lrgAB* expression by LytST is related to higher glucose availability during early exponential phase. Although detailed mechanistic studies have not yet been performed, there is mounting evidence that these proteins are critical for oxidative stress resistance in *S. mutans*: (1) *lrgAB* expression is highly regulated by oxygen ([11] and this study); (2) a *lrgAB* mutant was defective in aerobic growth on BHI agar plates [37]; (3) a *lrgAB* mutant displayed a decreased growth rate in the presence of paraquat (a superoxide-generating agent) relative to the wild-type strain [37]; and (4) a *lrgAB* mutant displayed a strong growth defect during static planktonic aerobic growth in BHI in the presence and absence of H_2_O_2_ challenge (this study). Interestingly, a link between LrgAB and oxidative stress was also demonstrated in *S. aureus*, where *lytSR* and *lrgAB* expression were upregulated 2-5 fold in response to azurophilic granule proteins, H_2_O_2_, and hypochlorite [54].

In agreement with a role for LrgAB in oxidative stress resistance, several LytST-regulated genes identified in this study have also been implicated in bacterial oxidative stress responses. Upregulated potential oxidative stress genes include *yghU*, a putative anti-oxidant enzyme [50], *tpx*, a predicted thiol peroxidase [55], and *recJ*, a single-stranded DNA exonuclease protein that facilitates DNA repair in response to oxidative stress [51]. Conversely, several genes belonging to the TnSMu2 gene cluster (SMU.1334c – SMU.1359) were downregulated in the *lytS* mutant. These genes are annotated as encoding a series of gene products involved in bacitracin and gramicidin synthesis [56], but more recently have been shown to be responsible for nonribosomal peptide and polyketide (NRP/PK) biosynthesis of a pigment that enhances aerobic growth and tolerance to H_2_O_2_ challenge in *S. mutans* UA159 [45]. The altered expression of one or more of these genes may explain, in part, the increased ROS accumulation that was observed in the *lytS* mutant when challenged with H_2_O_2_ (Figure 5). Furthermore, it was previously found that a two-component system responsible for positive regulation of the NRP/PK genes was located on the TnSMu2 genomic island of UA140 but not in UA159 [45]. This observation, combined with the microarray results performed here (Additional file 1: Table S1 and Additional file 2: Table S2) suggest that LytST may have taken over some of the regulatory functions of this non-core-genome two-component system that is missing in UA159.

Interestingly, H_2_O_2_ has also been shown to be a potent stimulator of competence and eDNA release in *S. sanguinis*[57], *S. gordonii*[57,58], and *S. pneumoniae*[59]. Although the effects of H_2_O_2_ on *S. mutans* competence, cell lysis, and eDNA release have not been directly measured, it has been shown that growth under aerobic conditions promotes competence in *S. mutans*[47], and that expression of competence-related genes is upregulated during aerobic growth [11]. The results presented here have demonstrated that expression of *comYB*, a gene encoding a component of the DNA-binding uptake system in *S. mutans*[47] was upregulated 2-fold in early exponential phase and 22-fold in late exponential phase in the *lytS* mutant (Additional file 1: Table S1 and Additional file 2: Table S2). The significance of high-level *comYB* expression in the *lytS* mutant at late exponential phase is unclear, given that maximal *S. mutans* competence develops in actively-growing populations [60,61]. Accordingly, upregulation of *comYB* expression did not correlate with increased transformability of the *lytS* mutant under the conditions tested in this study (Figure 3). However, it was found that the *lrgA* mutant displayed a significant reduction in competence. It has been recently reported that only a subpopulation of *S. mutans* culture lyses in response to CSP, and this lysis event is controlled in part by the CipB bacteriocin and the CipI immunity protein [62]. Subsequent microarray analysis of a *cipI* (immunity protein) mutant showed that both *lytST* and *lrgAB* expression were highly upregulated in the *cipI* mutant [63]. These results, combined with the fact that LrgA/B has been shown to be involved in regulating cell lysis and eDNA release in *S. aureus*[21,29], lends strong support to the idea that LrgA plays an important role during competence, possibly by altering membrane permeability or by modulating murein hydrolase activity.

The *S. mutans comY* operon consists of nine co-transcribed genes, of which the first eight genes are either essential to or significantly affect competence [46]. The ninth gene of this operon is predicted to encode acetate kinase (AckA), an enzyme that catalyzes the inter-conversion of acetyl-phosphate and acetate [46,64]. For micro-organisms with an inefficient or incomplete TCA cycle such as *S. mutans*, AckA-mediated conversion of acetyl-phosphate to acetate is thought to be a critical mechanism of generating ATP [reviewed in [65]]. Since *ackA* (*comYI*) was previously found to be upregulated in *S. mutans* during aerated growth [11], it is possible that LytST is involved in the regulation of energy generation through the phosphate acetyltransferase (Pta)-AckA pathway during aerobic growth and/or during oxidative stress. In this respect, it has recently been reported that an *S. mutans pta* mutant was more susceptible to both acid and oxidative stresses [66].

The ability of *S. mutans* to combat H_2_O_2_ stress is critical for its survival in the oral cavity, yet H_2_O_2_ detoxifying mechanisms and their regulation have not been extensively-characterized in this organism, limited primarily to the ScnRK and VicRK two-component systems [67,68], *ropA*[69], *brpA*[70], *luxS*[71] and genomic island TnSMu2 [45]. H_2_O_2_ has been shown to have potent antibacterial effects on *S. mutans*[72], and it is thought that H_2_O_2_ produced by other oral streptococcal species serves as an antagonist against *S. mutans*. For example, *S. sanguinis* and S*. gordonii* have been shown to produce H_2_O_2_ via pyruvate oxidase under aerobic growth conditions, and this H_2_O_2_ production allows them to compete effectively against *S. mutans* when co-cultured under aerobic growth conditions [57]. It is therefore possible that the *S. mutans* LytST regulon mediates a pleiotropic protective response against these H_2_O_2_-producing niche competitors. On-going and future studies by our group will focus on experimental testing of this hypothesis.

## Conclusions

In summary, the LytST two-component system has been shown to have a pleiotropic effect on gene expression in *S. mutans*. This is congruent with microarray analyses of *lytS* mutants in *S. aureus*[38] and *S. epidermidis*[40]. However, unlike in other organisms, we have been able to identify a pattern of LytS-mediated gene expression that suggests a role for this regulon in responding to oxidative/H_2_O_2_ stress. Although we have not yet been able to identify the external signal to which LytS responds, it is likely linked to an oxidative stress-sensing mechanism, such as H_2_O_2_-mediated membrane damage (ie. lipid peroxidation) via its large number of transmembrane domains, or oxygen/ROS interactions with its predicted cytoplasmic GAF domain, a ubiquitous signaling domain that has been shown to detect changes in the redox state of bound iron or oxygen in *Mycobacterium tuberculosis*[73-75]. Establishing mechanistic links between the LytST regulon, H_2_O_2_ resistance, and competence regulation will provide valuable new insights into *S. mutans* survival and virulence in the oral cavity.

## Methods

### Bacterial strains, media, and growth conditions

For all experiments, frozen glycerol stocks of *S. mutans* UA159 and its isogenic *lytS* (SAB111; Δ*lytS*::NPKm^r^), *lrgA* (SAB113; Δ*lrgA*::NPSp^r^), *lrgB* (SAB119; Δ*lrgB*::NPEm^r^), and *lrgAB* (SAB115; Δlrg*AB*::ΩKm^r^) mutants [created previously in [37] were freshly streaked for isolation on either Todd Hewitt Yeast Extract (THYE) or Brain Heart Infusion (BHI), containing selective antibiotic as appropriate: kanamycin (Km) – 1000 μg/ml, erythromycin (Em) – 10 μg/ml, spectinomycin (Sp) - 1000 μg/ml). With the exception of SAB115 (*lrgAB* mutant), all mutants were created using non-polar (NP) antibiotic-resistance markers [37]. Unless otherwise indicated, all *S. mutans* cultures were grown as static cultures in BHI or THYE broth at 37°C and 5% CO_2_.

### Analysis of *lrgAB* expression

To measure the effects of oxygen and glucose on *lrg* expression, overnight THYE cultures of UA159 and the *lytS* mutant (n = 3 biological replicates each, grown at 0 RPM, 37°C and 5% CO_2_) were each inoculated to an OD_600_ = 0.02 into THYE containing either 11 mM or 45 mM glucose. For “low O_2_” cultures, 2 L culture flasks each containing 400 ml media were grown at 0 RPM, 37°C, and 5% CO_2_. For aerobic cultures, 500 ml culture flasks each containing 100 ml media were grown at 37°C and 250 RPM. Total RNA was isolated from all cultures sampled at exponential (EP; OD_600_ = 0.2 – 0.4) and stationary (SP; OD600 = 1.4 – 1.7) growth phase, with an RNeasy Mini kit (Qiagen) and FASTPREP (MP Biomedicals) using previously-described methods [76]. Real-time reverse-transcriptase PCR and data analysis using *lrgA* and 16S primers was performed using previously described primers [37] and methods [77]. Fold-change expression of *lrgA* and 16S under each growth condition (11 mM low-O_2_, 11 mM aerobic, 45 mM low-O_2_, 45 mM aerobic) was calculated by dividing the gene copy number of each test sample by the average gene copy number of UA159 EP. Data was then normalized by dividing each *lrgA* fold-change expression value by its corresponding 16S fold-change expression value.

### RNA microarray analysis of UA159 and *lytS* mutant

To assess the effect of LytS on global gene expression, overnight BHI cultures of UA159 and *lytS* mutant (n = 3 biological replicates per strain) were diluted to an OD_600_ = 0.02 in BHI, and grown as static cultures at 37°C and 5% CO_2_. Total RNA was isolated from each culture at early-exponential (OD_600_ = 0.15) and late exponential phase (OD_600_ = 0.9), using previously-published methods [77]. RNA microarray analysis was performed using *S. mutans* UA159 microarrays provided by The Institute for Genomic Research, and previously-described methods and data analysis [11,70,78]. In brief, 2 μg total bacterial RNA was used in each reverse-transcription and cDNA labeling reaction (performed as described in [70,78]), and a single preparation from each culture was hybridized per microarray slide in a Maui hybridization chamber (BioMicro Systems, Salt Lake City, UT). The resulting microarray slides were scanned, analyzed, and normalized using TIGR Spotfinder software (http://www.tigr.org/software/), and in-slide replicate analysis was performed with the TIGR microarray data analysis system (MIDAS; http://www.tigr.org/software/). Statistical analysis was carried out with BRB array tools (http://linus.nci.nih.gov/BRB-ArrayTools.html/) with a cutoff *P* value < 0.005 for the early exponential-phase data and *P* < 0.001 for the late exponential phase data. To validate the microarray results, real-time quantitative RT-PCR was performed on a subset of the differentially-expressed genes, as described previously [77,79]. All real-time PCR primers were designed with Beacon Designer 4.0 software (Premier Biosoft International, Palo Alto, CA), and standard curves for each gene were prepared as published elsewhere [80]. The microarray data obtained from these studies have been deposited to NCBI’s gene expression omnibus (GEO) [81] (GEO Accession #GSE39470) and comply with MIAME guidelines [82].

### Quantitative competence assays

To compare the ability of UA159 and its isogenic *lytS*, *lrgA*, *lrgB*, and *lrgAB* mutants to take up exogenously-added plasmid DNA, a quantitative competence assay was performed on n = 4-6 biological replicates of each strain using a previously-published protocol [83] with the following modifications: Overnight cultures of each strain were diluted to an OD_600_ = 0.02 in BHI, and grown in a 96-well plate to an OD_600_ = 0.15 prior to addition of 500 ng plasmid DNA with and without 100 ng CSP. Plasmid pAT28 (encoding spectinomycin resistance; [84]) was used to assess transformation efficiency in UA159, *lytS*, *lrgB*, and *lrgAB* mutants. Because the *lrgA* mutant was generated with a spectinomycin-resistance cassette [37], plasmid pORi23 [encoding erythromycin resistance; [85]] was used to assess transformation efficiency in UA159 and *lrgA* mutant. After 2.5 h incubation in the presence of plasmid DNA +/- CSP, cultures were serially diluted and plated on BHI agar with and without selective antibiotic. CFU/ml of each culture were enumerated after 48 h growth at 37°C and 5% CO_2_, and transformation efficiencies were calculated as the percentage of transformants (CFU/ml on BHI + selective antibiotic) among total viable cells (CFU/ml on BHI).

### H_2_O_2_ assays

To assess of the ability of UA159, *lytS,* and *lrgAB* mutants to grow in the presence of H_2_O_2_, overnight cultures of each strain (n = 6 biological replicates) were each diluted 40-fold into BHI. 1 ml aliquots of each diluted culture were either untreated or challenged with 1 mM H_2_O_2_. Aliquots of each (500 μl per well, 2 wells total) were then immediately transferred to an optically-clear 48-well tissue culture plate (Costar 3548), which was incubated for 20 h at 37°C (aerobic atmosphere) in a Biotek Synergy microplate reader. OD_600_ measurements of each well were recorded at 2 h intervals.

### Oxidative stress measurements

To assess intracellular oxidative stress in UA159 and *lytS* mutant, single isolated colonies of each strain (n = 3-6 biological replicates per strain) were inoculated into culture tubes containing 4 ml BHI, and grown in “low-O_2_” conditions (37°C, 0 RPM, 5% CO_2_). After 20 h growth, 2 × 1 ml aliquots of each culture were harvested by centrifugation in a microcentrifuge (3 min at 13,000 RPM). The culture supernatants were discarded, and cell pellets were each resuspended in 1 ml Hanks Buffer (HBSS) containing 5 μM chloromethyl 2′,7′-dichlorofluorescein diaceate (CM-H_2_DCFDA; Invitrogen Molecular Probes), a cell-permeable fluorescent compound that is oxidized in the presence of H_2_O_2_ and other reactive oxygen species (ROS) and is considered a general indicator of cellular oxidative stress [52,53]. Cell suspensions were incubated at 37°C for 60 min to “load” the cells with CM-H_2_DCFDA, followed by centrifugation (3 min at 13,000 RPM). Supernatants were discarded, and cell pellets were washed once with HBSS prior to resuspension in 1 ml HBSS or in 1 ml HBSS containing 5 mM H_2_O_2_. Each cell suspension was transferred into triplicate wells (200 μl per well) of an optically-clear 96 well plate (Costar 3614), and the plate was transferred to a Biotek Synergy microplate reader. Fluorescence in relative fluorescence units (RFU; using 492-495 nm excitation and 517-527 nm emission) and OD_600_ readings of each well were recorded after 30 min incubation at 37°C.

### Statistical analysis

All statistical analyses, unless otherwise indicated, were performed using Sigmaplot for Windows 11.0 software (Build 11.0.0.75, Systat Software, Inc.).

## Competing interests

The authors declare that they have no competing interests.

## Authors' contributions

SJA carried out the RNA microarray experiments and associated data analysis, performed all real-time PCR studies, participated in the conception and design of the study, and helped draft the manuscript. MDQ carried out all of the RNA isolations for comparing the effects of glucose and oxygenation on *lrgAB* expression. ER optimized and carried out all of the quantitative competence assays. RAB participated in the design and coordination of the study, and helped draft the manuscript. KCR participated in the conception and design of the study, performed the H_2_O_2_ assays, intracellular ROS measurements, and drafted the manuscript. All authors read and approved the final manuscript.

## Supplementary Material

Additional file 1 **Table S1. **Genes differentially expressed by loss of LytS at early-exponential phase (P< 0.005). Click here for file

Additional file 2 **Table S2. **Genes differentially expressed by loss of LytS at late exponential phase (P< 0.001). Click here for file
