# Supplementary material for: Identification of the Streptococcus mutans LytST two-component regulon reveals its contribution to oxidative stress tolerance
Source: BMC Microbiol. 2012 Sep 1;12:187. doi: 10.1186/1471-2180-12-187 (PMC3507848; doi:10.1186/1471-2180-12-187)
Supplement: Additional file 1 — Table S1. Genes differentially expressed by loss of LytS at early-exponential phase (P< 0.005). [file 1471-2180-12-187-S1.docx]

Table S1. Genes differentially expressed by loss of LytS at early-exponential phase (*P*< 0.005)

| **Functional group** | **Gene symbol** | **Description** | **Fold-change (*lytS*/wild-type)** |
| --- | --- | --- | --- |
| **Cellular processes** | | | |
| *Chaperones* |  |  |  |
| SMU.956c | *clpL, clpE* | putative Clp-like ATP-dependent protease, ATP-binding subunit | 1.6060089 |
| SMU.1955c | *groES* | co-chaperonin GroES | 0.7375962 |
|  |  |  |  |
| *Pathogenesis* |  |  |  |
| SMU.1396 | [*gbpC*](http://www.ncbi.nlm.nih.gov/entrez/query.fcgi?cmd=search&db=gene&term=gbpC) | glucan-binding protein C, GbpC | 0.6540701 |
|  |  |  |  |
| *Protein and peptide secretion* | | | |
| SMU.589 |  | putative DNA-binding protein | 0.7800888 |
|  |  |  |  |
| *Toxin production and resistance* | | | |
| SMU.1339 | [*bacD*](http://www.ncbi.nlm.nih.gov/entrez/query.fcgi?cmd=search&db=gene&term=bacD) | putative bacitracin synthetase | 0.0302466 |
| SMU.1340 | *bacA2* | putative surfactin synthetase | 0.0750438 |
| SMU.1341c |  | putative gramicidin S synthetase | 0.0120763 |
| SMU.1342 | [*bacA1*](http://www.ncbi.nlm.nih.gov/entrez/query.fcgi?cmd=search&db=gene&term=bacA1) | putative bacitracin synthetase 1, BacA | 0.021351 |
|  |  |  |  |
| **DNA metabolism** |  |  |  |
| SMU.1967 | *ssbA* | single-stranded DNA-binding protein | 1.3516108 |
|  |  |  |  |
| **Energy metabolism** |  |  |  |
| *Sugars* |  |  |  |
| SMU.1004 | *gtfB* | glucosyltransferase-I | 0.5220086 |
|  |  |  |  |
| **Fatty acid and phospholipid metabolism** | | | |
| SMU.1335c |  | putative enoyl-(acyl-carrier-protein) reductase | 0.6312763 |
| SMU.1344c |  | putative malonyl-CoA acyl-carrier-protein transacylase | 0.0077153 |
|  |  |  |  |
| **Hypothetical** |  |  |  |
| SMU.55 |  | hypothetical protein | 1.2114844 |
| SMU.618 |  | hypothetical protein | 0.8050277 |
| SMU.1360c |  | hypothetical protein | 0.2861994 |
|  |  |  |  |
| **Mobil and extrachromosomal element functions** | | | |
| SMU.767 |  | putative transposase | 0.7201993 |
| SMU.1354c |  | putative transposase | 0.3185797 |
| SMU.1363c | *tpn* | putative transposase | 0.0875149 |
| SMU.1379 | *tpn* | putative transposase | 0.4902598 |
|  |  |  |  |
| **Protein fate** |  |  |  |
| SMU.539c | *hopD, comC* | signal peptidase type IV | 1.8597887 |
|  |  |  |  |
| **Purines, pyrimidines, nucleosides, and nucleotides** | | | |
| SMU.32 | *purF, purB* | amidophosphoribosyltransferase | 1.3723331 |
| SMU.34 | *purM* | phosphoribosylaminoimidazole synthetase | 1.3693086 |
| SMU.35 | *purN* | phosphoribosylglycinamide formyltransferase | 1.4040296 |
|  |  |  |  |
| **Signal transduction** | | | |
| *PTS* |  |  |  |
| *SMU.1957c* | *levG, ptnD* | putative PTS system, mannose-specific IID component | 0.7169777 |
| *SMU.1958c* | *levF* | putative PTS system, mannose-specific IIC component | 0.7095883 |
| *SMU.1960c* | *levE* | putative PTS system, mannose-specific IIB component | 0.6800983 |
|  |  |  |  |
| *Two-component systems* |  |  |  |
| *SMU.577* | *lytS* | putative histidine kinase LytS | 0.0214838 |
|  |  |  |  |
| **Transport and binding proteins** | | | |
| SMU.1365c | *ylbB* | permease | 0.0184631 |
| SMU.1366c |  | putative ABC transporter, ATP-binding protein | 0.025025 |
| SMU.1985 | [*comYB*](http://www.ncbi.nlm.nih.gov/entrez/query.fcgi?cmd=search&db=gene&term=comYB) | ABC transporter ComYB | 2.2386703 |
| SMU.1987 | [*comYA*](http://www.ncbi.nlm.nih.gov/entrez/query.fcgi?cmd=search&db=gene&term=comYA) | putative ABC transporter, ATP-binding protein ComYA, late competence gene | 2.1892837 |
|  |  |  |  |
| **Unassigned** |  |  |  |
| SMU.574c | *lrg* | lrgB-like family protein | 0.4989436 |
| SMU.575c | *lrgA* | holin-like protein LrgA | 0.484686 |
| SMU.1345c |  | putative peptide synthetase MycA | 0.024107 |
| SMU.1346 | *bacT* | putative thioesterase BacT | 0.0131318 |
|  |  |  |  |
| **Unknown** | | | |
| SMU.53 |  | Conserved hypothetical protein | 1.2504286 |
| SMU.1327c |  | Conserved hypothetical protein, 4Fe-4S binding domain | 0.5220086 |
| SMU.1349 |  | Conserved hypothetical protein | 2.0185992 |
| SMU.1956c |  | Conserved hypothetical protein | 0.7068102 |
| SMU.1982c |  | Conserved hypothetical protein | 2.044082 |
|  |  |  |  |
