# Supplementary material for: Identification of the Streptococcus mutans LytST two-component regulon reveals its contribution to oxidative stress tolerance
Source: BMC Microbiol. 2012 Sep 1;12:187. doi: 10.1186/1471-2180-12-187 (PMC3507848; doi:10.1186/1471-2180-12-187)
Supplement: Additional file 2 — Table S2. Genes differentially expressed by loss of LytS at late exponential phase (P< 0.001). [file 1471-2180-12-187-S2.docx]

Table S2. Genes differentially expressed by loss of LytS at late exponential phase (*P*< 0.001)

| **Functional group** | **Gene symbol** | **Description** | **Fold-change (*lytS*/wild-type)** |
| --- | --- | --- | --- |
| **Amino acid biosynthesis** | | | |
| *Pyruvate family* |  |  |  |
| SMU.233 | *ilvC* | ketol-acid reductoisomerase | 3.6018586 |
| SMU.1023 | *pycB* | oxaloacetate decarboxylase | 0.2798236 |
| SMU.157 | *cysE* | putative serine acetyltransferase, serine O-acetyltransferase | 1.8680243 |
|  |  |  |  |
| **Biosynthesis of cofactors, prosthetic groups, and carriers** | | | |
| SMU.1296 | *yghU* | putative glutathione S-transferase YghU | 8.961394 |
| SMU.1084 | *hemK* | putative protoporphyrinogen oxidase | 2.2752837 |
| SMU.582 | *ispA, fps* | putative farnesyl diphosphate synthase | 0.4890623 |
| SMU.841 |  | putative aminotransferase | 2.077611 |
|  |  |  |  |
| **Cell envelope** |  |  |  |
| SMU.1688 | *dltD* | putative extramembranal protein, DltD protein | 2.7890333 |
| SMU.455 | *pdp2x* | putative penicillin-binding protein 2X | 2.4351256 |
| SMU.1039c | *waaR, waaJ, kdt* | putative lipopolysaccharide glycosyltransferase | 0.3236446 |
|  |  |  |  |
| **Cellular processes** | | | |
| *Adaptation to atypical conditions* | | | |
| SMU.924 | *tpx* | thiol peroxidase | 2.4148275 |
|  |  |  |  |
| *Cell division* |  |  |  |
| SMU.15 | *ftsH* | putative cell division protein FtsH | 2.0504131 |
| SMU.1003 | *gid* | tRNA (uracil-5-)-methyltransferase Gid | 1.7732082 |
| SMU.713 | *ftsW* | putative cell division protein FtsW | 1.7183926 |
| SMU.1324 | *ftsX* | putative cell-division protein FtsX | 2.6479981 |
|  |  |  |  |
| *Chaperones* |  |  |  |
| SMU.1954 | *groEL* | chaperonin GroEL | 0.5289494 |
| SMU427 | *copZ* | putative copper chaperone | 0.3809382 |
| SMU.82 | *dnaK* | molecular chaperone DnaK | 0.1523051 |
|  |  |  |  |
| *Detoxification* |  |  |  |
| SMU.1286c | *yitG, blt* | putative permease, multidrug efflux protein | 3.5693367 |
|  | | | |
| *Toxin production and resistance* | | | |
| SMU.1339 | *bacC* | putative bacitracin synthetase | 0.0193854 |
| SMU.1342 | *bacA1* | putative bacitracin synthetase 1, BacA | 0.0128161 |
| SMU.1341c | *grs, mycB* | putative gramicidin S synthetase | 0.0086318 |
| SMU.1340 | *bacA2* | putative surfactin synthetase | 0.0307009 |
|  |  |  |  |
| **Central intermediary metabolism** | | | |
| SMU.636 | *nagB* | putative N-acetylglucosamine-6-phosphate isomerase | 3.0522759 |
| SMU.1322 | *budC* | acetoin reductase | 1.7732552 |
| SMU.1687 | *ppaC* | putative manganese-dependent inorganic pyrophosphatase | 1.7436224 |
|  |  |  |  |
| **DNA metabolism** |  |  |  |
| SMU.1967 | *ssbA* | single-stranded DNA-binding protein | 5.5803232 |
| SMU.1472 | *recJ* | putative single-strand DNA-specific exonuclease RecJ | 4.5589829 |
|  |  |  |  |
| **Energy metabolism** |  |  |  |
| *ATP-proton motive force interconversion* | | | |
| SMU.1528 | *atpB* | F0F1 ATP synthase subunit beta | 2.582669 |
| SMU.1527 | *atpC* | F0F1 ATP synthase subunit epsilon | 2.1918756 |
| *Biosynthesis and degradation of polysaccharides* | | | |
| SMU.1432c | *bgc* | putative endoglucanase precursor | 2.1643653 |
| *Fermentation* |  |  |  |
| SMU.1424 | *pdhD* | putative dihydrolipoamide dehydrogenase | 0.4769444 |
| SMU.1423 | *pdhA* | putative pyruvate dehydrogenase, TPP-dependent E1 component alpha-subunit | 0.4088209 |
| SMU.1421 | *pdhC* | branched-chain alpha-keto acid dehydrogenase subunit E2 | 0.1532662 |
| SMU.1011 | *citG* | putative CitG protein | 0.2764563 |
|  |  |  |  |
| *Glycolysis/gluconeogenesis* |  |  |  |
| SMU.99 | *fbaA* | fructose-bisphosphate aldolase | 2.4059349 |
| SMU.113 | *pfk* | putative fructose-1-phosphate kinase | 0.1597532 |
| SMU.1489 | *lacX* | LacX | 0.1118628 |
| *Sugars* |  |  |  |
| SMU.887 | *galT* | galactose-1-phosphate uridylyltransferase | 0.4829715 |
| SMU.1535 | *phsG* | glycogen phosphorylase | 0.3388341 |
| SMU.495 | *gldA* | glycerol dehydrogenase | 0.1858757 |
| SMU.1490 | *lacG* | 6-phospho-beta-galactosidase | 0.1020705 |
|  |  |  |  |
| **Fatty acid and phospholipid metabolism** | | | |
| SMU.1739 | *fabF* | 3-oxoacyl-(acyl carrier protein) synthase II | 2.2443808 |
| SMU.962 | *mmgC* | putative dehydrogenase | 2.7906811 |
| SMU.1734 | *accA* | acetyl-CoA carboxylase subunit alpha | 2.660529 |
| SMU.1735 | *accD* | acetyl-CoA carboxylase subunit beta | 2.2984387 |
| SMU.1344c | *fabD* | putative malonyl-CoA acyl-carrier-protein transacylase | 0.0223155 |
|  |  |  |  |
| **Hypothetical** |  |  |  |
| SMU.958 |  | Hypothetical protein | 4.4670806 |
| SMU.1587c |  | Hypothetical protein | 2.8223919 |
| SMU.2105 |  | Hypothetical protein | 2.365655 |
| SMU.614 |  | Hypothetical protein | 2.1191022 |
| SMU.1946 |  | Hypothetical protein | 1.941314 |
| SMU.1360c |  | Hypothetical protein | 0.1569922 |
|  |  |  |  |
| **Mobile and extrachromosomal element functions** | | | |
| SMU.1354c |  | putative putative transposase | 0.2418496 |
| SMU.1363c | *tpn* | putative transposase | 0.0278486 |
|  |  |  |  |
| **Protein fate** |  |  |  |
| *Degradation of proteins, peptides, and glycopeptides* | | | |
| SMU.395 | *pepX* | x-prolyl-dipeptidyl aminopeptidase | 0.4433766 |
|  |  |  |  |
| *Protein folding and stabilization* |  |  |  |
| SMU.83 | *dnaJ* | heat shock protein DnaJ (HSP-40) | 0.2918908 |
| SMU.81 | *grpE* | heat shock protein GrpE (HSP-70 cofactor) | 0.2638283 |
|  |  |  |  |
| *Protein modification and repair* |  |  |  |
| SMU.755 | *lgt* | prolipoprotein diacylglyceryl transferase | 1.7790999 |
|  |  |  |  |
| **Protein synthesis** |  |  |  |
| *Protein modification* |  |  |  |
| SMU.1051 | *nifS* | iron-sulfur cofactor synthesis protein | 0.5142172 |
|  | | | |
| *Ribosomal proteins: synthesis and modification* | | | |
| SMU.2000 | *rplQ* | 50S ribosomal protein L17 | 6.3196395 |
| SMU.2002 | *rs11* | 30S ribosomal protein S11 | 4.9743901 |
| SMU.2003 | *rpsM* | 30S ribosomal protein S13 | 4.7967458 |
| SMU.2003c | *rpmJ* | 50S ribosomal protein L36 | 4.3377373 |
| SMU.957 | *rplJ* | 50S ribosomal protein L10 | 3.7847357 |
| SMU.960 | *rplL* | 50S ribosomal protein L7/L12 | 3.5678142 |
| SMU.2032 | *rpsB* | 30S ribosomal protein S2 | 3.1319002 |
| SMU.169 | *rplM* | 50S ribosomal protein L13 | 2.9645129 |
| SMU.170 | *rpsI* | 30S ribosomal protein S9 | 2.9612505 |
| SMU1626 | *rplA* | 50S ribosomal protein L1 | 2.6580663 |
| SMU.2026c | *rps10, rpsJ* | 30S ribosomal protein S10 | 2.2157606 |
| SMU.1200 | *rpsA* | 30S ribosomal protein S1 | 2.2048536 |
| SMU.1627 | *rplK* | 50S ribosomal protein L11 | 2.2026091 |
|  |  |  |  |
| *Translation factors* |  |  |  |
| SMU.2004 | *infA* | translation initiation factor IF-1 | 3.1577867 |
| SMU.359 | *fus, tetO* | elongation factor G | 2.1290956 |
| *Other* |  |  |  |
| SMU.1606 | *smpB* | SsrA-binding protein | 2.1903478 |
|  |  |  |  |
| *tRNA aminoacylation* |  |  |  |
| SMU.1311 | *asnC* | asparaginyl-tRNA synthetase | 2.2366516 |
| SMU.1770 | *valS* | valyl-tRNA synthetase | 2.1066599 |
| SMU.1586 | *thrS* | threonyl-tRNA synthetase | 1.9637127 |
| *Other* |  |  |  |
| SMU.1606 | *smpB* | SsrA-binding protein | 2.1903478 |
|  |  |  |  |
| **Purines, pyrimidines, nucleosides, and nucleotides** | | | |
| SMU.944 | *thyA* | thymidylate synthase | 3.1234637 |
| SMU.1086 | *kith* | thymidine kinase | 2.2807132 |
| SMU.580 | *xseA* | exodeoxyribonuclease VII large subunit | 0.2949093 |
|  |  |  |  |
| **Regulatory functions** |  |  |  |
| *DNA interactions* |  |  |  |
| SMU.80 | *hrcA* | heat-inducible transcription repressor | 0.4953722 |
| SMU.953c |  | putative transcriptional regulator/aminotransferase | 0.4070108 |
| SMU.584 | *argR, ahrC* | putative arginine repressor | 0.3546557 |
|  |  |  |  |
| General regulatory |  |  |  |
| SMU.105 | *scrR, lacI* | putative transcriptional regulator, repressor of sugar transport operon | 0.490465 |
|  |  |  |  |
| *Other* |  |  |  |
| SMU.1515 | *covX* | hypothetical protein | 2.6764309 |
|  |  |  |  |
| **Signal transduction** |  |  |  |
| *PTS* |  |  |  |
| SMU.1600 | *celB* | cellobiose phosphotransferase system IIB component | 0.218699 |
| SMU.114 |  | putative PTS system, fructose-specific IIBC component | 0.1936115 |
| SMU.115 |  | putative PTS system, fructose-specific IIA component | 0.1914331 |
| SMU.1598 | *celC* | cellobiose phosphotransferase system IIA component | 0.1738983 |
| SMU.1596 | *celD* | cellobiose phosphotransferase system IIC component | 0.1633683 |
|  |  |  |  |
| *Two-component systems* |  |  |  |
| SMU.1129 | *ciaR* | putative response regulator CiaR | 0.4817681 |
| SMU.1037c | *phoR* | putative histidine kinase | 0.3980582 |
| SMU.577 | *lytS* | putative histidine kinase LytS | 0.0048108 |
|  |  |  |  |
| **Transcription** |  |  |  |
| SMU.611 | *deaD, rheA* | ATP-dependent RNA helicase | 2.2659316 |
| SMU.2001 | *rpoA* | DNA-directed RNA polymerase subunit alpha | 5.5173626 |
| SMU.1745c |  | putative transcriptional regulator | 2.763516 |
| SMU.1599 | *celR* | putative transcriptional regulator, possible antiterminator | 0.1755442 |
|  |  |  |  |
| **Transport and binding proteins** | | | |
| SMU.568 |  | putative amino acid ABC transporter, ATP-binding protein | 2.3434772 |
| SMU.1666 | *livG* | putative branched chain amino acid ABC transporter, ATP-binding protein | 2.1183644 |
| SMU.1325 | *ftsE* | putative ABC transporter, ATP-binding component | 1.7886366 |
| SMU.1366c |  | putative ABC transporter, ATP-binding protein | 0.0185912 |
| SMU.1668 | *livH* | putative branched chain amino acid ABC transporter, permease protein | 2.264163 |
| SMU.1667 | *livM* | putative branched chain amino acid ABC transporter, permease protein | 2.004054 |
| SMU.879 | *msmF* | multiple sugar-binding ABC transporter, permease protein MsmF | 0.4471718 |
| SMU.1365c | *ylbB* | Permease | 0.0161324 |
| SMU.1963 | *levQ* | putative sugar-binding periplasmic protein | 0.4323656 |
| SMU.880 | *msmG* | multiple sugar-binding ABC transporter, permease protein MsmG | 0.4254579 |
| SMU.1985 | *comYB* | ABC transporter ComYB | 22.9927435 |
|  |  |  |  |
|  |  |  |  |
| **Unassigned** |  |  |  |
| SMU.367 |  | Streptococcus-specific protein; similar to glucan-binding protein | 1.8993877 |
| SMU.1511c |  | putative acetyltransferase; ; possible transcriptional repressor | 1.8718513 |
| SMU.587 |  | lipase/acylhydrolase with GDSL-like domain | 0.5174147 |
| SMU.400 |  | putative secreted esterase | 0.4787778 |
| SMU.1040c | *ydfG* | putative oxidoreductase, short-chain dehydrogenase/reductase | 0.4780691 |
| SMU.583 | *hlyX* | putative hemolysin | 0.2493708 |
| SMU.1345c | *ituA* | putative peptide synthetase MycA | 0.1495599 |
| SMU.1346 | *bacT* | putative thioesterase BacT | 0.0393511 |
| SMU.575c | *lrgA* | holin-like protein LrgA | 0.0049531 |
| SMU.574c | *lrg* | lrgB-like family protien | 0.004215 |
|  |  |  |  |
| **Unknown** |  |  |  |
| SMU.1545c |  | Conserved hypothetical protein | 4.7357545 |
| SMU.508 |  | Conserved hypothetical protein, possible hydrolase | 4.4179662 |
| SMU.509 |  | Conserved hypothetical protein | 3.7447991 |
| SMU.2046c |  | Conserved hypothetical protein | 3.0173063 |
| SMU.1506c |  | Conserved hypothetical protein, possible multidrug efflux pump | 3.0151856 |
| SMU.516 |  | Conserved hypothetical protein, N6 adenine-specific DNA methylase signature domain | 2.9309334 |
| SMU.260 |  | Conserved hypothetical protein | 2.7516912 |
| SMU.159 |  | Conserved hypothetical proteinRibonuclease III family protein | 2.6082643 |
| SMU.1083c |  | Conserved hypothetical protein | 2.5998184 |
| SMU.1621c |  | Conserved hypothetical protein | 2.2656552 |
| SMU.239c |  | Conserved hypothetical protein, putative transporter | 2.0467063 |
| SMU.1623c |  | Conserved hypothetical protein, predicted s1 RNA binding domain | 1.8833143 |
| SMU.298 |  | Conserved hypothetical protein, predicted CoA-binding protein | 1.7995944 |
| SMU.588 |  | Conserved hypothetical protein | 0.3923297 |
| SMU.1022 | *citX2* | Conserved hypothetical protein, possible CitXG protein | 0.2912856 |
|  |  |  |  |
